# Co-evolution of Friendships and Antipathies: A Longitudinal Study of Preschool Peer Groups

**DOI:** 10.3389/fpsyg.2016.01509

**Published:** 2016-10-06

**Authors:** João R. Daniel, António J. Santos, Marta Antunes, Marília Fernandes, Brian E. Vaughn

**Affiliations:** ^1^William James Center for Research, ISPA—Instituto UniversitárioLisbon, Portugal; ^2^Human Development and Family Studies, Auburn UniversityAuburn, AL, USA

**Keywords:** preschool children, peer groups, friendships, antipathies, stochastic actor-based models, multivariate analysis

## Abstract

We used stochastic actor-based models to test whether the developmental dynamics of friendships and antipathies in preschool peer groups (followed throughout three school years) were co-dependent. We combined choices from three sociometric tasks of 142 children to identify friendship and antipathy ties and used SIENA to model network dynamics. Our results show that different social processes drive the development of friendship and antipathy ties, and that they do not develop in association (i.e., friendship ties are not dependent on existing antipathies, and vice-versa). These results differ from those of older children (age range = 10–14) suggesting that the interplay of friendship and antipathy only plays a significant role in the peer group context in older children. We propose these differences to be likely related with preschool age children's inaccurate perceptions of their classmates' relationships, particularly of their antipathies, and/or with the absence of shared norms to deal with antipathetic relationships.

## Introduction

Although ties within classroom peer groups can be broadly classified in two types—positive (e.g., friendship, support, cooperation) and negative (e.g., antipathy, bullying)—most studies focus on ties with a positive valence and neglect the role of negative ties in children's social lives. Only recently have researchers started to focus on the interplay between both types of ties (Huitsing et al., 2012, 2014; Berger and Dijkstra, 2013; Rambaran et al., 2015); but to our knowledge no study addressing how positive and negative ties develop together in preschool peer groups has been published, to date. Because preschool children have limited experience of complex networks, the preschool period provides an ideal context to study the basic processes behind relationship formation (Snyder et al., 1996; Olson and Spelke, 2008). Studying these processes is of the upmost importance given that the structures of social networks are likely the result of an adaptive process that promotes social development by creating an environment where uncertainty of others behavior is reduced (van den Oord et al., 2000; Flack et al., 2013).

### Positive ties

Positive interactions with peers have long been recognized as essential to social adaptation throughout childhood and adolescence (Rubin et al., 2006; Vaughn and Santos, 2009; Vaughn et al., 2016). Recently, developmental studies of peer relationships have benefited from advances in longitudinal social network analysis. For instance, Schaefer et al. (2010) used stochastic-actor based models (see Snijders et al., 2010 for an introduction to these models) to show that the creation of friendship ties (inferred from observational data) in preschool children is influenced by the ubiquitous sex homophily effect (Martin and Ruble, 2009; Martin et al., 2013), in combination with reciprocity, in-degree popularity, and triadic closure effects (see also Daniel et al., 2013 for similar evidence using cross-sectional data). In other words, Schaefer et al. (2010) observed that throughout the school-year, preschool children tended to create sex segregated (sex homophily effect) and reciprocal preferences (reciprocity effect), to concentrate their preferences in peers that were also preferred by several others (in-degree popularity effect), and to befriend their friends' friends (triadic closure effect).

Similar effects have been obtained for samples of older children and adolescents using the same network modeling approach (see for example Block and Grund's (2014) analysis of 11 secondary and middle schools in Scotland, England, Wales, and United States), supporting early socio-ethological claims that socialization effects are observed at any age, as long as children are inserted in stable peer groups (Strayer, 1980). But while social network studies of school-age children and adolescents have increased exponentially, studies of preschool children remain scarce. One of the reasons for this is that studies of older children tend to use (time-friendly) self-report (sociometric) methods to identify interpersonal relationships, while studies of the preschool children mostly tend to use (time-consuming) observational methods. Although there are some criticisms regarding the use of sociometric tasks to collect network information of preschool children (Strayer, 1980), social network analysis of preschool sociometric data suggests that this type of data is valid for studying developmental aspects of young children' relationships (van den Oord et al., 2000; see also Shin et al., 2014 for evidence showing that sociometric data can yield valid assessments of peer friendships).

The first goal of our study was thus to try to replicate Schaefer et al.'s findings using friendship data inferred from sociometric data rather than from observational data (Schaefer et al., 2010 used the number of times children were observed interacting together to identify friendship ties). Because sociometric peer preferences and observed peer associations reflect a dynamic social process (Snyder et al., 1996) we expect to replicate Schaefer et al.'s (2010) findings. Namely, we hypothesize that with time children tend to create (or maintain):

(H1) reciprocal friendships (reciprocity effect);
(H2) friendship ties with peers that are preferred by several others (in-degree popularity effect);
(H3) friendships with the friends of their friends (triadic closure effect);
(H4) same sex friendships (sex homophily effect).

Our predictions are not only supported by Schaefer et al.'s (2010) findings but also on extensive research (on human and non-human species) showing that that reciprocity, in-degree popularity, triadic closure and homophily effects are ubiquitous in networks of positive relationships (Faust and Skvoretz, 2002). While reciprocity is a basic feature of preschool dyadic relationships that emerges early in childhood (Snyder et al., 1996; Paulus and Moore, 2012; Paulus, 2016), in-degree popularity is likely to result from children seeking others based upon behavioral characteristics that are unevenly distributed among peers (Schaefer et al., 2010), like sociability, sensitivity or play style (Gifford-Smith and Brownell, 2003). Triadic closure is likely to emerge in preschool children as a consequence of increased propinquity between children who share mutual friends (Schaefer et al., 2010). By observing the pro-social interactions of their friends, children are likely to increase their propensity to act pro-socially toward these new third-parties (Olson and Spelke, 2008) and later create new friendship relations. Lastly, sex homophily is not only the result of behavioral compatibility between same-sex children, but also the consequence of created expectations that same-sex children share similar interests (Martin et al., 2013).

### Negative ties

Within the scope of negative ties, antipathies (i.e., ties based on dislike) relate with important development outcomes. High frequencies of antipathies are associated with a number of maladjustment problems (e.g., externalizing and internalizing problems, low academic achievement, low prosocial behavior, victimization and rejection by peers, and lower social preference and friendships; see review by Card, 2010). Nevertheless, Witkow and colleagues have shown that, when controlling for rejection and analyzing only adolescents who receive at least one rejection nomination, having a mutual antipathy does not necessarily associate with increased maladjustment (Witkow et al., 2005).

Recent studies (Huitsing et al., 2012; Berger and Dijkstra, 2013; Rambaran et al., 2015) have highlighted the relational nature of antipathies (i.e., an active dislike of specific peers results from an interactional process) and the consequent advantages of studying the developmental processes of these ties from a social network perspective; instead of just looking at rejection as an individual characteristic (i.e., number of dislike choices received by peers in a sociometric task). Little is known about the developmental dynamics of antipathy ties in preschool children. Thus, the second goal of our study was to model for the first time the development of antipathy ties in preschool children and compare it to that of friendship ties.

Our expectation is that the developmental dynamics of both types of ties differ similarly to what has been observed in older samples (Berger and Dijkstra, 2013; Huitsing et al., 2014; Rambaran et al., 2015). We hypothesize that with time children tend to create (or maintain):

(H5) antipathy ties with peers that are also disliked by several others (in-degree popularity effect);
(H6) cross sex antipathies (sex heterophily effect); but not:
(H7) reciprocal antipathies;
(H8) antipathies that form closed triads.

Our predictions are based on sociometric (Hayes, 1978; Hayes et al., 1980; van den Oord et al., 2000) studies showing that dislike sociometric choices are less stable, less likely to be reciprocated, less transitive, and concentrated in fewer children than like choices. Also, dislike choices tend to fall on members of the opposite sex, contrary to the same-sex tendency found for like choices (see also Fujisawa et al., 2009 for similar findings from observational data).

### Multivariate ties

Multivariate (or multiplex) networks are social networks where nodes are connected by different types of ties (here, friendships and antipathies). The simultaneous study of positive and negative ties stems from balance theory (Heider, 1946; see also Cartwright and Harary, 1956; Newcomb, 1961). Balance theory claims that if actors perceive that their ties create tension or imbalance, they change them in order to reach a balanced state, creating patterns of ties perceived as comfortable and stable. Signed ties (i.e., ties that can be positive or negative) are balanced if a positive tie between two actors is consistent with their negative ties with the third member of the triad. So far, balance theory has been used to explain the interplay of friendship and antipathies in elementary school children and young adolescents (Huitsing et al., 2012; Berger and Dijkstra, 2013; Rambaran et al., 2015). Whether balance is also seen for preschool children is still unknown.

**Figure 1:**
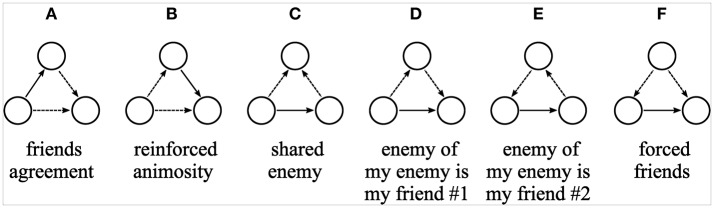
**Balanced triads**. Graphical representations illustrating different processes through which the creation (or maintenance) of the tie presented in the base of the triangle creates (or maintains) balance. Solid lines represent friendship ties and dotted lines antipathy ties. Arrow heads indicate who is directing the tie toward whom.

Figure 1 illustrates six hypothetical processes through which positive and negative ties create (or maintain) a balanced triad. Although all triads represented are balanced, they are created differently. In Figures 1A,B balance is achieved by creating (or maintaining) an antipathy that matches a friend's dislike (“friends agreement”), or by creating (or maintaining) an antipathy with a friend of a child one dislikes (“reinforced animosity”). In the remaining figures balance is achieved by creating (or maintaining) a friendship. Like Figures 1A,C (“shared enemy”) depicts a triad where friends agree on whom they dislike, but in this case, balance is achieved when children that share the same antipathy create (or maintain) a friendship. In Figure 1D balance is achieved when children create (or maintain) a friendship with peers who are disliked by the children they dislike, whereas in Figure 1E a friendship is created (or maintained) in response to a received negative tie. We grouped both Figures 1D,E under the same label (“enemy of my enemy is my friend”) because in both a friendship is created (or maintained) in response to an indirect negative tie. Finally, in the last triadic configuration Figure 1F (“forced friends”) friendships are created (or maintained) between children who are disliked by the same others.

But while balance theory is ubiquitously used to explain the interplay of positive and negative ties, recent findings suggest that this interplay might be better explained by a combination of different processes (balance included; Yap and Harrigan, 2015). From the set of eight theories reviewed by Yap and Harrigan (2015), expected to influence the formation of signed ties, we highlight here status theory and visibility theory (as for balance theory, these two theories also assume that positive and negative ties are co-dependent). Instead of dealing with triadic relationships (as balance does), status and visibility deal with degree related effects. That is, these theories claim that the number of existing (positive and negative) ties are used as clues as to whether someone is a desirable partner.

Status theory (Leskovec et al., 2010) posits that an individual's likelihood of receiving positive (negative) ties increases (decreases) with higher status (with status referring to the difference between received positive and negative ties). In other words, according to status theory, with time, the number of received positive and negative ties should become negatively correlated (i.e., more ties of one type leads to less of the other). On the other hand, visibility theory (Yap and Harrigan, 2015), posits that an individual's likelihood of receiving positive or negative ties will be proportional to their total number of received positive and negative ties. In other words, according to visibility theory, with time, the number of received positive and negative ties should become positively correlated.

Another possibility for co-dependence of positive and negative ties deals with a positive correlation between positive and negative ties appearing as a general response tendency in nominating others (reflected both in like and dislike choices; Huitsing et al., 2012). This response resembles Yap and Harrigan's (2015) activity theory, but in this case, extended to multivariate ties. Yap and Harrigan (2015) only considered the hypothesis that individual's likelihood of sending a new positive (negative) tie increases with the number of positive (negative) ties they have already sent (i.e., positive ties only influence positive ties, and negative ties only influence negative ties), but not the hypothesis that the likelihood of sending a new positive or negative tie increases with the number of positive and negative ties.

Like balance, these three theories can be tested within the framework of stochastic actor based models (using multivariate degree related effects, instead of triadic effects used to test balance). The predictive power of these theories to explain developmental dynamics of friendship and antipathetic ties in preschool children has never been tested before. The third goal of our study was thus to test whether balance, status, visibility, and activity explain the co-development of friendships and antipathies in preschool children. Based on these theories, we hypothesize that with time children tend to create (or maintain):

(H9) multivariate triads as predicted by balance theory (Figure 1);
(H10) either a negative correlation between the number of received positive and negative ties as predicted by status theory or (H11) a positive correlation as predicted by visibility theory;
(H12) a positive correlation between the number of sent positive and negative ties as predicted by activity theory.

One important difference between balance, and visibility and status theories concerns the cognitive demands they imply (higher for balance). While, balance theory implies that children perceive friendship and antipathies of the peers they like and dislike, and act accordingly (to create or maintain balance), status and visibility theories only require that children have a general knowledge about which peers are more salient in their classroom (i.e., which peers are more liked and disliked). Contrary to balance, status, or visibility, activity theory does not imply that the co-dependency between friendship and antipathy ties arises as a consequence of children creating or changing their ties according to what they perceive to be the relationships of their peers. If co-dependency occurs is simply the consequence of a general response tendency to send friendship and antipathy ties. Due to these differences in complexity we expect the effects with less cognitive demands to be more relevant.

Social network evidence of multivariate degree related effects from elementary school children and young adolescents is scarce and inconclusive. While, Rambaran et al.'s (2015) longitudinal study did not find evidence for multivariate degree related effects influencing the co-development of friendships and antipathies, Huitsing et al.'s (2012) cross sectional study supported the degree correlations predicted by status theory—the number of like and dislike choices received was negatively related. Previous sociometric studies, showing that the number of like and dislike choices received are negatively correlated (e.g., Wasik, 1987, reference withheld for blind review) suggest that status might play a role in the development of friendships and antipathies in preschool children as well. Also the fact that only a small proportion of children achieve a controversial status in sociometric studies (i.e., children that receive several like and disliked choices; e.g., Terry and Coie, 1991) suggests that visibility effects might not be very relevant within preschool peer groups.

## Materials and methods

### Participants

Different pairs of research assistants familiar to the children collected sociometric data in five different preschool classrooms, in two centers serving middle class families in the Lisbon area (Portugal), in 3 consecutive years (once a year between January and June). Classrooms size ranged from 20 to 28 “same-age” children (*M* = 24.27): either “3-years-old” (i.e., children < 48 months of age at the start of the academic year; wave 1), “4-years-old” (i.e., children between 48 and 60 months of age at the start of the academic year; wave 2) or “5-years-old” (i.e., children between 60 and 72 months of age at the start of the academic year; wave 3). Participation rates ranged between 72 and 100% (*M* = 89%). Seventy-seven children participated in all three data waves, 28 children in two and 37 children only in one, for a total of 142 different children (65 girls). Between 72 and 96% (*M* = 84%) of same classroom children transited together from one academic year to the next. Table 1 presents descriptive information for the five classrooms.

**Table 1:** **Network composition**.

**Classroom**	***N* = ♂, + ♀**			**Leavers/Joiners**	
	**Wave 1**	**Wave 2**	**Wave 3**	**Period 1**	**Period 2**
C1	9 ± 11	9 ± 12	12 ± 11	5/6	5/7
C2	12 ± 13	9 ± 11	13 ± 12	8/3	1/6
C3	11 ± 7	16 ± 7	12 ± 8	0/5	5/2
C4	11 ± 8	13 ± 8	12 ± 7	1/33	4/2
C5	13 ± 12	10 ± 12	12 ± 11	3/0	1/2
Total	56 ± 51	57 ± 50	61 ± 49	17/17	16/19

*Network size (N = 142) equals the number children present at least in one wave; wave 1, “3-year-olds”; wave 2, “4-year-olds”; wave 3, “5-year-olds”; period- time between waves; leavers- number of children that leave a specific classroom in the end of the school year; joiners- number of children that join a specific classroom in the beginning of a new school year; values refer only to children who participated in the study (non-authorized children are excluded)*.

Written consent for children's participation was obtained from school directors, teachers, and parents prior to data collection. The project was approved by the Portuguese Data Protection Authority (CNPD, n° 1379/08).

### Procedure

Children completed three picture sociometric tasks (administered individually in a quiet room) in the following order: nominations (McCandless and Marshall, 1957), rating scale (Asher et al., 1979), and paired comparisons (Starkweather, 1962). These tasks took between 30 and 45 min to complete (usually in two sessions). For the nominations task, children were presented with photographs of all classmates and asked to name a peer with whom they especially liked to play; the request was repeated two more times (total of three like nominations). After, children were asked to identify a peer with whom they did not especially like to play; this request was also repeated two more times. For the rating scale task, children rated the photographs of all classmates by placing them in one of three containers: children with whom they liked to play a lot, sort of liked to play, or did not like to play with (scored 3, 2, and 1, respectively). Beforehand, children were trained to use the scale by being asked to rate the degree to which they liked certain food items (chocolate, sandwich, tomato). For the paired comparisons task, photographs of all the possible pairs in each classroom [i.e., *N* (*N* − 1)/2 pairs] were presented to children. For each pair, children were asked to choose the peer with whom they especially liked to play. The pairs were randomly organized, and no child was seen twice before all classmates were seen once. Each child's photograph appeared the same number of times on the left- and right-hand sides of the picture file.

### Friendship and antipathy ties

Following Vaughn et al. (2000) we combined the choices from the three sociometric tasks to identify friendships. We considered a friendship tie to exist (total = 1214, *M* per children = 3.90, *SD* = 1.66, all waves considered) if a child gave another peer a rating-scale score of “3” (Rat3 = 2987, *M* = 9.60, *SD* = 3.80), and that peer was either among the top 20% of her/his choices on the paired comparisons task (PC+ = 1448, *M* = 4.79, *SD* = 1.03), or was one of her/his three positive nominations (Nom+ = 930). In a similar fashion, we considered an antipathy to exist (total = 870, *M* = 2.80, *SD* = 1.77) if a child gave another peer a rating-scale score of “1” (Rat1 = 1729, *M* = 5.54, *SD* = 3.38), and that peer was either among the bottom 20% of her/his choices on the paired comparisons task (PC^−^ = 1507, *M* = 5.00, *SD* = 1.17), or was one of her/his three negative nominations (Nom^−^ = 930). Agreement between the different sociometric tasks was higher for like choices than for dislike choices (Table 2).

**Table 2:** **Agreement between sociometric tasks for like and dislike choices**.

	**% of Agreement**		
	**Wave 1 (%)**	**Wave 2 (%)**	**Wave 3 (%)**
**LIKE CHOICES**			
Nom+ and Rat3	65	66	83
PC+ and Rat3	62	68	79
**DISLIKE CHOICES**			
Nom− and Rat1	37	46	49
PC− and Rat1	38	46	48

*Nom+, positive nomination; Rat3, rating score of 3; PC+, choice in the top 20% of paired comparisons task; Nom−, negative nomination; Rat1, rating score of 1; PC−, choice in the bottom 20% of paired comparisons task*.

As detailed in Vaughn et al. (2000), this combination of sociometric tasks to identify friendships and antipathies: (1) corrects for the problem of underestimating the number of friends/antipathies when only a limited number of nominations are specified, (2) does not fix an arbitrary number of choices that may overestimate the number of friends/antipathies, and (3) is more reliable because nominations and paired-comparisons choices have to match a score of 3 (or 1) on the rating-scale.

### Analytic strategy: rsiena

We used actor-based Simulation Investigation for Empirical Network Analysis (SIENA) in R (Ripley et al., 2015) to model the network dynamics of friendship and antipathy ties (R script available at withheld for blind review). SIENA actor-based modeling framework is more thoroughly detailed in Snijders et al. (Snijders et al. (2010), see also Snijders et al., 2013 for an introduction to multivariate models). These models represent network dynamics as being determined by different effects (e.g., reciprocity, homophily) that operate simultaneously. In doing so, they allow to test for the presence of these effects and to estimate parameters expressing their strength. These parameters are analogous to regression coefficients in (logistic) regression and indicate the importance of each effect (“predictor variables”) in creating (or maintaining) a tie (“dependent variable”; 1, tie; 0, no tie).

Due to the small sample sizes of our networks, we arranged our data as one large matrix with structural zeroes between children in different classrooms (i.e., one large network where ties between children in different classrooms are not possible). Although this approach prevented us from identifying unique processes of specific classrooms, it circumvented convergence problems (i.e., low reliability of estimates) when trying to fit models to individual classrooms.

We also used structural zeros to account for classroom composition changes over time (i.e., children joining and leaving classrooms at the beginning or the end of the school year, respectively; see Table 1 for counts of joiners and leavers). Structural zeros were specified for all ties toward and from children who were absent at a given observation wave.

**Figure 2:**
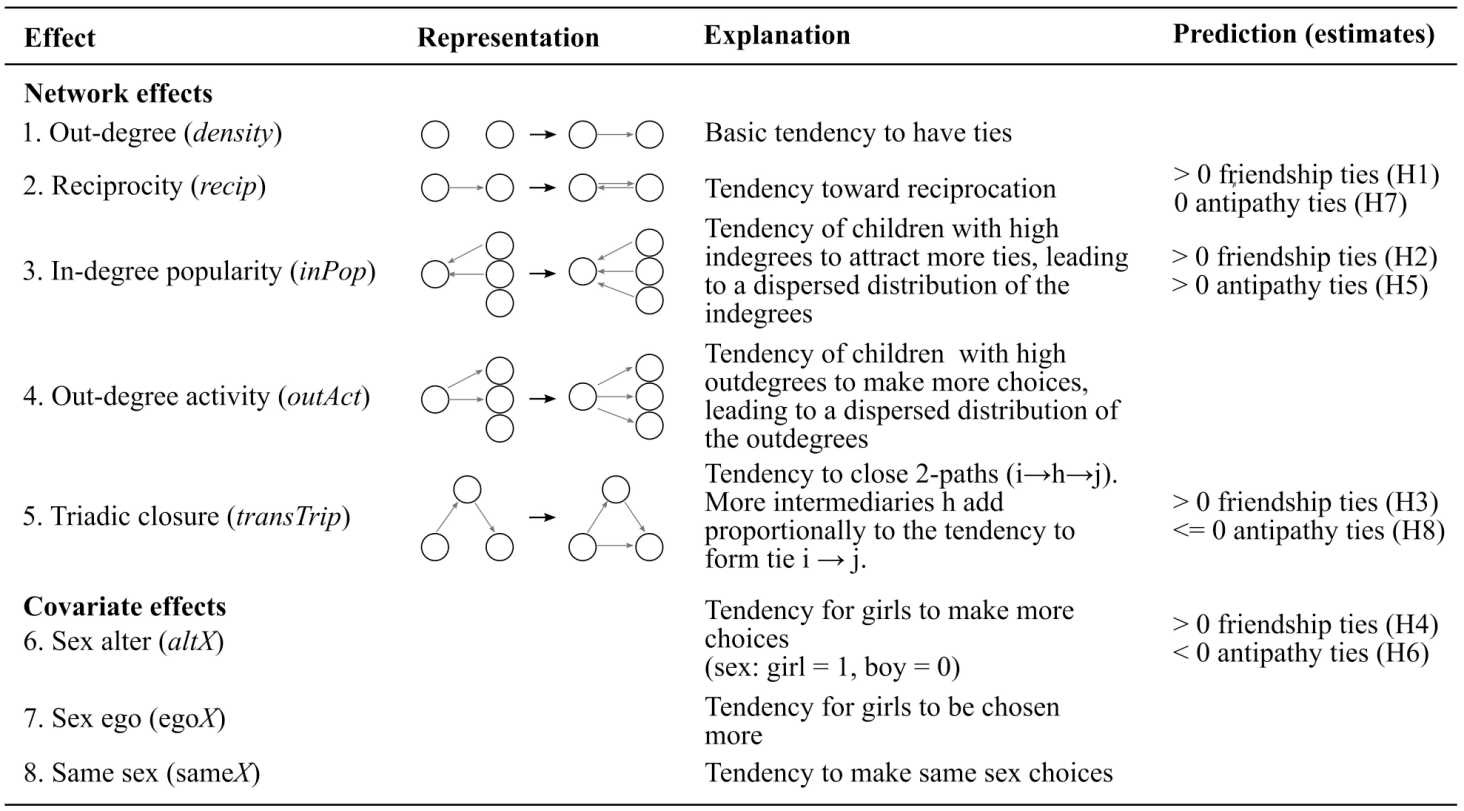
**Summary of SIENA univariate effects**. If the predicted estimate equals 0, the corresponding effect is expected to play no role in the network dynamics; if positive, the formation (or maintenance) of ties that create the corresponding representation are expected to occur; and the converse if the predicted estimate is negative. For detailed explanations concerning these predictions please see Sections Positive Ties and Negative Ties. Out-degree effect (average tendency to form ties) can be interpreted similarly to the intercept of regression models; out-degree activity effect was included *a posteriori* to improve model fit, and ego and alter sex covariate effects are included to control for possible sex differences in the number of ties.

**Figure 3:**
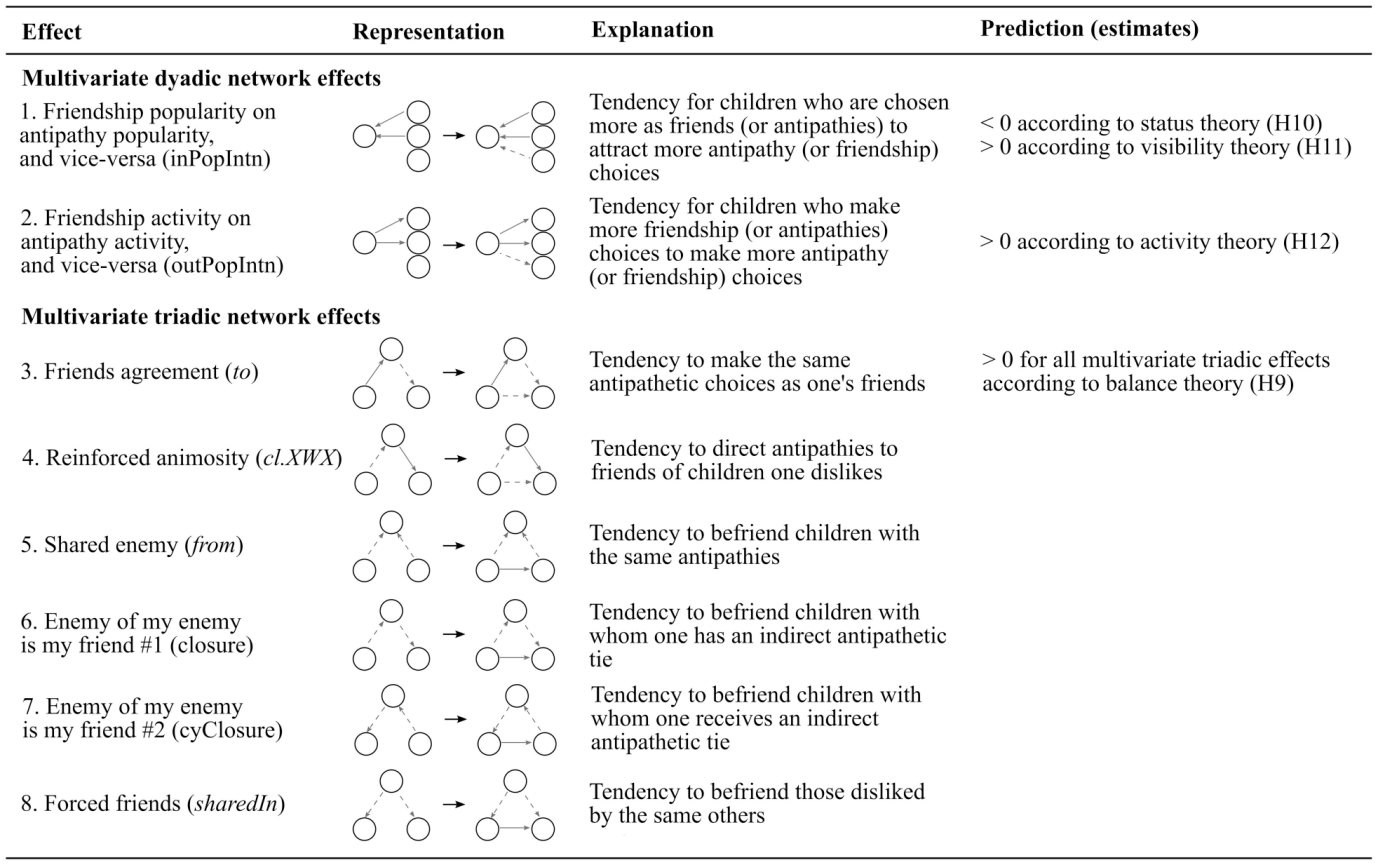
**Summary of SIENA multivariate effects**. Solid lines represent friendship ties and dotted lines antipathy ties. If the predicted estimate is positive, the formation (or maintenance) of ties that create the corresponding representation are expected to occur; and the converse if the predicted estimate is negative. For detailed explanations concerning these predictions please see Section Multivariate Ties.

Figures 2, 3 present a visual representation of all effects included in the models as well as the key predictions hypothesized in Sections Positive Ties to Multivariate Ties. Before estimating the multivariate model we estimated univariate models separately for friendship and antipathy networks. All models included three sex (covariate) effects (sex alter, sex ego, and same sex). The choice of network effects followed the general guidelines provided by Ripley et al. (2015) and includes effects that allowed us to test our predictions concerning the formation (and maintenance) of friendships and antipathies. Besides the effects used to test our hypothesis we included the basic out-degree effect (effect 1, Figure 2; average tendency to form ties; similar to the intercept of regression models), and ego and alter sex covariate effects (effects 6 and 7, Figure 2) to control for possible sex differences in the number of ties. Out-degree activity effect (effect 2, Figure 2) was included *a posteriori* to improve model fit.

To help the interpretation of model estimates, particularly when comparing univariate friendship and antipathy models, we calculated the expected relative importance of each effect (Indlekofer and Brandes, 2013). This statistic is analogous to an effect size measure and captures the influence of each effect on actor's decisions of creating or maintaining ties (the sum of the expected relative importance of all effects included in a model equals 1).

Also, we conducted time heterogeneity tests, to determine whether the effects' estimates changed from wave to wave (if time heterogeneity exists it may be evidence of model misspecication; Lospinoso et al., 2011), and goodness of fit tests to see how well the model reproduced features (auxiliary network statistics) of the observed data not explicitly fit in the model (Lospinoso, 2012).

## Results

### Comparison of the univariate models

Table 3 presents descriptive statistics of the friendship and antipathy networks, while Table 4 presents parameter estimates of the univariate models that allows us to interpret which effects underlie the dynamics of these networks. There was no time heterogeneity (i.e., effects were stable across time; *p*-values of the joint significance test of time heterogeneity were *ns*), no convergence problems (i.e., estimates were reliable; overall maximum convergence ratios < 0.25, mean absolute individual *t* statistics < 0.10), and goodness-of-fit was acceptable for both models (i.e., included effects were sufficient to explain network dynamics; Supplementary Figures 1, 2). Comparison of the parameter estimates and the expected relative importance of each effect (Table 4) reveals substantial differences between friendship and antipathy network dynamics confirming our predictions.

**Table 3:** **Descriptive statistics of friendship and antipathy networks**.

	**Friendship network**						**Antipathy network**				
	**Wave 1**		**Wave 2**		**Wave 3**		**Wave 1**		**Wave 2**		**Wave 3**
Missing fraction	0.007		0.006		0.002		0.007		0.006		0.002
*M* degree	3.79		3.61		4.25		2.67		2.79		2.89
Proportion of reciprocated ties	0.35		0.33		0.43		0.16		0.13		0.17
Proportion of same gender ties	0.57		0.62		0.72		0.42		0.38		0.16
♀/♂*M* in-degree	3.39/3.75		3.24/3.61		3.88/4.55		2.45/2.59		2.44/2.86		3.29/2.57
♀/♂*M* out-degree	3.92/3.68		3.87/3.40		4.21/4.28		2.50/2.83		2.84/2.75		2.75/3.00
SD in-degree	2.16		2.38		3.18		2.21		2.36		3.49
CV in-degree	0.60		0.69		0.75		0.87		0.89		1.21
SD out-degree	1.83		1.75		1.36		1.99		1.70		1.64
CV out-degree	0.48		0.48		0.32		0.74		0.61		0.57
**TIE CHANGES**											
Creating tie (0 → 1)		263		306				241		253
Dissolving tie (1 → 0)		269		233				206		236
Stable tie (1 → 1)		94		135				44		49
Jaccard index		0.15		0.20				0.09		0.09

*Wave 1, “3-year-olds”; wave 2, “4-year-olds”; wave 3, “5-year-olds”; Jaccard index (stability between two waves) = (1 → 1)/(0 → 1+1 → 0 + 1 → 1)*.

**Table 4:** **Univariate models: parameter estimates (β), standard errors (*SE*), and expected relevance importance for each wave (R W)**.

	**Friendship network**					**Antipathy network**				
	**β**	***SE***	**R W1**	**R W2**	**R W3**	**β**	***SE***	**R W1**	**R W2**	**R W3**
Rate (period 1)	12.37^*^	1.45				13.50	4.13			
Rate (period 2)	10.23^*^	1.05				9.96	1.31			
**NETWORK EFFECTS**										
1. Out-degree	−0.52	0.36	0.10	0.10	0.09	−1.79^*^	0.15	0.18	0.17	0.15
2. Reciprocity	0.64^*^	0.10	0.04	0.04	0.04	0.16	0.15	0.01	0.01	0.00
3. In-degree popularity	0.04^*^	0.02	0.16	0.16	0.18	0.19^*^	0.02	0.47	0.49	0.54
4. Out-degree activity	−0.17^*^	0.04	0.14	0.14	0.14	0.05	0.03	0.06	0.07	0.06
5. Triadic closure	0.30^*^	0.04	0.07	0.08	0.12	−0.30^*^	0.13	0.02	0.02	0.03
**COVARIATE EFFECTS**										
6. Sex(F) alter	−0.05	0.06	0.04	0.04	0.04	−0.02	0.08	0.02	0.01	0.01
7. Sex(F) ego	0.15	0.09	0.02	0.02	0.02	−0.12	0.09	0.02	0.02	0.01
8. Same sex	0.43^*^	0.07	0.43	0.43	0.37	−0.31^*^	0.10	0.23	0.21	0.19

*Rate effects model the expected opportunities actors had to change their outgoing ties between successive waves; estimates of network and covariate effects parameters are analogous to regression coefficients in (logistic) regression and indicate the importance of each effect in creating (or maintaining) ties; expected relative importance statistic captures the influence of each effects on actor's decisions on creating or maintaining ties (because the sum of the expected relative importance of all effects included in a model equals 1, this statist resembles and effect size measure); overall maximum convergence ratio: friendship network = 0.23, antipathy network = 0.10; mean absolute t statistics for deviations from targets: friendship network = 0.02, antipathy network = 0.02; joint significance test of time heterogeneity—friendship network: χ^2^ = 13.32, df = 8, p = 0.10, antipathy network: χ^2^ = 11.4, df = 8, p = 0.19*.

* *p < 0.05*.

Reciprocity effects were positive in both networks (Table 4, effect 2: friendship β = 0.64, antipathy β = 0.16) but only statistically significant for friendship ties. Meaning that through time children tended to create or maintain reciprocal friendships (H1), but not reciprocal antipathies (H7) (% reciprocal ties wave 1–3: friendship = 35, 33, and 43%, antipathy = 16, 13, and 17%; Table 3).

Positive and significant in-degree popularity effects were present in both networks (Table 4, effect 3: friendship β = 0.04, antipathy β = 0.19). Meaning that children chosen more often (as friends or antipathies) were more likely to attract additional ties through time (H2 and H5), but the relative importance of this effect was much higher for antipathies (the highest of all antipathy effects; relevance wave 1–3: friendship = 0.16, 0.16, and 0.18, antipathy = 0.47, 0.49, and 0.54; *CV* in-degree: friendship = 0.60, 0.69, and 0.75, antipathy = 0.87, 0.89, and 1.21; Table 3).

Out-degree activity effect was only significant for friendship ties (Table 4—effect 4: friendship β = −0.17, antipathy β = 0.05), indicating a more homogeneous friendship out-degree distribution (*CV* out-degree wave 1–3: friendship = 0.48, 0.48, and 0.32, antipathy = 0.74, 0.61, and 0.57; Table 3).

Triadic closure effect was significant in both networks but with inverse signs (Table 4—effect 5: friendship β = 0.30, antipathy β = −0.30), indicating that through time children tended to become friends of their friends (H3), but not to dislike those that are disliked by the ones they dislike (H8).

Same-sex effect estimates (Table 4—effect 8) confirmed the important role of sex in the dynamics of friendship (H4) and antipathy ties (H6). The same-sex effect was the most relevant effect in the friendship network (relevance wave 1–3: friendship = 0.43, 0.43, and 0.47, antipathy = 0.23, 0.21, and 0.19), but while children tended to befriend same-sex partners (β = 0.43) they tended to dislike (β = −0.31) opposite sex partners (% same sex ties wave 1–3: friendship = 57, 62, and 72%, antipathy = 16, 13, and 17%; Table 3). Sex alter (Table 4—effect 6: friendship β = −0.05, antipathy β = −0.02,) and sex ego effects (Table 4—effect 7: friendship β = 0.15, antipathy β = −0.12) indicate that when controlling for other effects boys and girls were equally likely to choose or be chosen by others as friends or antipathies.

Jaccard indices (i.e., tie stability between two waves; formula given on the bottom of Table 3) found for both networks indicate a substantial rearrangement of ties from one year to the next, with antipathies (Jaccard index = 0.09 for period 1 and 2; i.e., from wave 1 to wave 2, and from wave 2 to wave 3 9% of ties were maintained) being less stable than friendships (period 1 = 0.15, period 2 = 0.20). Although a Jaccard index of at least 0.20 is recommended to use SIENA actor-based models (Snijders et al., 2010) this had no consequence for model convergence (i.e., parameter estimates reliability; overall maximum convergence ratios < 0.25 and mean absolute individual *t* statistics < 0.10 for all models). Because the Jaccard index is influenced by the number of joiners and leavers in classrooms at the end of each school year, we computed this index exclusively for children that were present in all three waves. For the friendship network these values were 0.21 and 0.31 for period 1 and 2, respectively, and 0.11 for the antipathy network for both periods.

### Multivariate model

Estimates for the multivariate model are presented in Table 5. These estimates allow us to interpret whether friendships and antipathies develop in association. As in the univariate models, there was no time heterogeneity (i.e., effects were stable across time), no convergence problems (i.e., estimates were reliable), and goodness-of-fit was acceptable (included effects were sufficient to explain network dynamics; Supplementary Figures 3, 4). Considering the fact that contrary to balance (H9), status (H10), visibility (H11), and activity theory (H12) predictions none of the multivariate effects (degree-related or triadic) was statistically significant (Table 5), multivariate effects do not appear to be determinant for the development of friendship and antipathy ties in preschool children. In other words, we did not find evidence that friendship and antipathy ties were mutually influencing (i.e., they did not co-evolve within the group).

**Table 5:** **Multivariate model: parameter estimates (β), standard errors (*SE*) and expected relevance importance for each wave (R W)**.

**Effect**	**β**	***SE***	**R W1**	**R W2**	**R W3**
**FRIENDSHIP**					
Rate (period 1)	12.75^*^	2.24			
Rate (period 2)	10.32^*^	1.53			
**Friendship Network Effects**					
1. Out-degree	−0.04	0.66	0.00	0.00	0.00
2. Reciprocity	0.62^*^	0.14	0.02	0.02	0.02
3. In-degree popularity	−0.12	0.13	0.18	0.18	0.19
4. Out-degree activity	−0.17^*^	0.04	0.08	0.09	0.09
5. Triadic closure	0.28^*^	0.09	0.04	0.04	0.07
**Friendship Covariate Effects**					
6. Sex(F) alter	−0.07	0.11	0.03	0.03	0.02
7. Sex(F) ego	0.17	0.14	0.01	0.01	0.01
8. Same sex	0.38^*^	0.11			
**ANTIPATHY**					
Rate (period 1)	12.81^*^	3.19			
Rate (period 2)	11.25^*^	2.77			
**Antipathy Network Effects**					
9. Out-degree	−0.95	0.63	0.10	0.10	0.10
10. Reciprocity	0.12	0.28	0.00	0.00	0.00
11. In-degree popularity	−0.04	0.13	0.06	0.06	0.07
12. Out-degree activity	−0.07	0.08	0.05	0.05	0.05
13. Triadic closure	−0.01	0.19	0.00	0.00	0.00
**Antipathy Covariate Effects**					
14. Sex(F) alter	−0.08	0.10	0.04	0.04	0.04
15. Sex(F) ego	−0.11	0.14	0.01	0.01	0.01
16. Same sex	−0.17	0.09	0.09	0.09	0.09
**MULTIVARIATE**					
**Degree-Related Network Effects**					
17. Friendship popularity on antipathy popularity (−)	−0.31	0.21	0.59	0.57	0.57
18. Antipathy popularity on friendship popularity (+)	−0.36	0.33	0.38	0.37	0.36
19. Friendship activity on antipathy activity (−)	0.03	0.19	0.01	0.01	0.01
20. Antipathy activity on friendship activity (+)	−0.12	0.16			
**Triadic Network Effects**					
21. Friends agreement (−)	0.23	0.31	0.02	0.02	0.02
22. Reinforced animosity (−)	0.48	0.27	0.03	0.04	0.04
23. Shared enemy (+)	0.17	0.31	0.04	0.04	0.06
24. Enemy of my enemy #1 (+)	0.45	1.16	0.03	0.03	0.04
25. Enemy of my enemy #2 (+)	0.19	0.44	0.02	0.02	0.02
26. Forced friends (+)	0.37	0.33	0.01	0.01	0.01

Rate effects model the expected opportunities actors had to change their outgoing ties between successive waves; expected relative importance statistic captures the influence of each effects on actor's decisions on creating or maintaining ties (the sum of the expected relative importance of all effects included in the model equals 1 for friendship and antipathy effects separately); + and − signs following multivariate effects' names indicate whether friendship (+) or antipathy (−) ties were considered the dependent tie; overall maximum convergence ratio = 0.20; mean absolute t statistics for deviations from targets = 0.02; joint significance test of time heterogeneity: χ^2^ = 35.00, df = 26, p = 0.11;

* *p < 0.05*.

Although the friendship popularity on antipathy popularity effect, and the antipathy popularity effect on friendship popularity effects had high expected relevance they were non-significant. To see if any of the multivariate effects reached statistical significance in a simpler model (fewer effects, less likely to occur collinearity problems, and more likely to obtain significant effects) we decided to explore additional multivariate models including just univariate and sex effects (Table 5—effects 1–16), plus only one of the multivariate effects at a time (Table 5—effects 17–26). None was found (Supplementary Table 1).

## Discussion

The aim of this study was to model the developmental dynamics of friendship and antipathy ties in preschool classrooms and test whether friendships and antipathies co-evolve together. Our findings show that different processes determine the development of ties in both networks, replicating previous findings from older children (Berger and Dijkstra, 2013; Rambaran et al., 2015). The combination of friendship and antipathy networks in a multivariate model did not support the presence of balance, nor degree-related dependencies between both networks has found in older children.

### Friendship ties

The first goal of our study was to try to replicate previous findings for preschool children using friendship data inferred from sociometric data instead of observational data (Schaefer et al., 2010). Following our expectations, results show that reciprocity (mutual preference), in-degree popularity (preference for more central peers), triadic closure (preference for friends of friends), and sex homophily (preference for same-sex peers) jointly contributed to changes in the pattern of friendship ties, similarly to what has been reported by Schaefer et al. This replication supports the validity of using sociometric data to study the development of friendship relations in preschool children using a social network approach. Had the reliability of sociometric data been a major problem, we would have been unable to find similar social processes to those described in Schaefer et al.'s observational study. Our results also agree with the idea suggested by Faust and Skvoretz (2002) that reciprocity, in-degree popularity and triadic closure effects are quite ubiquitous in networks of positive relationships.

Despite Jaccard indices (period 1 = 0.15, period 2 = 0.20) indicating that there was a substantial rearrangement of friendship from 1 year to the next, the amount of change was similar to that reported in studies of older children where sociometric data was also collected once a year. For instance, Berger and Dijkstra's (2013) study of fifth- and sixth-graders reports a Jaccard index of 0.19 (they only collected data for two waves, so only one period exists), while Rambaran et al.'s (2015) study of sixth-, seventh-, and eighth-graders reports a Jaccard index of 0.18 and 0.22 (periods 1 and 2, school 1), and of 0.13 and 0.18 (school 2). In the case of Rambaran et al. (2015), the authors only used data of children who participated in all 3 years of data collection. The Jaccard indices calculated for our sample in this manner (including only children present in all 3 years of data collection) is even higher (period 1 = 0.21, period 2 = 0.32) than that reported by Rambaran et al. (2015). In addition, non-published data reference withheld for blind review, including children from this sample, indicate that the stability of relationships inferred from observational data is similar to that reported here. Although these results highlight that peer relationships in preschool children have a significant degree of fluidity (Poulin and Chan, 2010), it does not appear to be much different than that of older children, independently of the type of data used (sociometric or observational).

### Antipathy ties

As expected, results from the antipathy univariate model agree with previous descriptions of observed interactions and distribution of sociometric (dislike) nominations in preschool children (Hayes, 1978; Hayes et al., 1980; van den Oord et al., 2000; Fujisawa et al., 2009). Negative networks (derived from observational data) have fewer and smaller cliques than networks of positive relationships; and dislike nominations are concentrated in fewer children than like nominations, and less likely to be reciprocated. These findings fit with the significant positive in-degree popularity effect (i.e., disliked peers became more disliked over time), significant negative triadic closure effect (i.e., lack of clique like structures), and non-significant reciprocity effect. Briefly, with time children tended to direct their disliked choices to cross-sex peers and to dislike those that were mostly disliked by their peers.

Like in the friendship network, sex had a determining role in the development of antipathy ties: antipathy ties were more common between cross-sex peers (increasing from 58% at wave 1 to 84% at wave 3). These results (together with the sex homophily found for the friendship network) support the ubiquity of sex-segregated patterns of social interaction and its central role in the social organization of preschool peer groups (Martin and Ruble, 2009; Martin et al., 2013).

Reciprocity effect was non-significant. The proportion of reciprocated antipathies ranged between 13 and 17% (wave 1–3), compared to 33–43% of reciprocated friendships. Hayes (1978) suggested that the lack of reciprocity of disliking indicates that strong mutual animosities are highly transitory and seldom exist among young children. The substantial rearrangement of antipathy ties from 1 year to the next (low Jaccard indices, 0.09 for both periods) and the low levels of reciprocity (between 13 and 17% in the three waves) support Hayes' claims. Nevertheless, this does not seem to be exclusive of preschool children; similar low reciprocity levels and (in)stability has been described in older children (Berger and Dijkstra, 2013: reciprocity ~20%, Jaccard index = 0.13; Rambaran et al., 2015: reciprocity ~10%, Jaccard indices ranging from 0.06 to 0.09).

Although antipathetic ties were highly transitory, children who were commonly disliked by others with time tended to increase their level of rejection. Thus, the establishment and maintenance of a negative social reputation agrees with van den Oord et al.'s (2000) arguments that antipathetic choices generally reflect a group assessment of individual “problem” children that likely incur in aggression, rule violation, and aberrant behavior (Hayes, 1978; Hayes et al., 1980).

In-degree popularity effect is the effect most consistently found in studies that modeled the dynamics of antipathy ties. Other effects are not as consistently found as in studies of friendship networks. For example, we did not found evidences of triadic closure effects in antipathies, while Rambaran et al. (2015) did (Berger and Dijkstra, 2013 did not test univariate triadic effects for antipathies); nor reciprocity effects, while Rambaran et al. and Berger and Dijkstra did. Negative same sex effects were present in our study and in Rambaran et al. (2015) but not in Berger and Dijkstra (2013). Whether these differences relate to different network sizes and densities (mean number of antipathies: our study, ~3; Berger and Dijkstra, 2013, ~2; Rambaran et al., 2015, ~8), to the different combination of effects used in the models, or to differences in sociometric tasks remains to be tested.

Univariate network and sex antipathy effects were less robust (than friendship effects) to the inclusion of multivariate network effects in the model. Also, the agreement between dislike choices from the three sociometric tasks (we used to infer antipathy ties) was moderate (~40%), and much lower to that found between like choices (~70%; Table 2). This indicates that children had more difficulties in identifying the peers they disliked than the ones they liked. But again, lower agreement for dislike choices in sociometric tasks is not exclusive of preschool children; similar findings have been reported for elementary and junior high school children (Maassen et al., 1997). Maassen and colleagues even report lower levels of agreement for high school than for elementary school children. As such, even though the agreement level was moderate, the combination of sociometric tasks we used to identify antipathies at least gives us some confidence that the antipathy ties reflect true dislike relationships. Similar studies with older children have only resorted to one sociometric task to identify friendships or antipathies (Huitsing et al., 2012; Berger and Dijkstra, 2013; Rambaran et al., 2015). Hayes (1978) and Rekalidou and Konstantinos (2012) have shown that when asked to justify their choices, preschool children tend to provide less criteria for their dislike choices. Thus, lower agreement for dislike sociometric choices might reflect a more general human difficulty of giving finer evaluative distinctions when conveying attitudes about liked vs. disliked objects (Smallman et al., 2014), but that it is not exclusive of preschool children.

### Multivariate ties

We did not found evidence that friendship and antipathy ties co-develop in association, either through degree-related effects (status, visibility, activity) or triadic effects (balance). In other words, the developmental dynamics of friendship and antipathy networks were independent of one another—existing friendship relationships did not drive the formation of antipathies or vice-versa. This result differs from previous findings in older children where multivariate triadic effects have been found to create (or maintain) balance triads (Berger and Dijkstra, 2013; Rambaran et al., 2015; see also Huitsing et al., 2012 for evidence of balance in a cross-sectional study), thus indicating that friendship and antipathetic relations are not co-dependent for this age sample (age range = 10–14).

Status and visibility theory (Leskovec et al., 2010; Yap and Harrigan, 2015) assume that the number of existing (positive and negative) ties serve as clue as to whether someone is a desirable partner. According to status theory, with time, the number of received positive and negative ties should become negatively correlated (i.e., more friendships, less antipathies, and vice-versa), while visibility theory predicts the opposite (with time, the number of the number of received positive and negative ties should become positively correlated). We did not find evidence supporting either status or visibility, or even an activity process, wherein the co-dependence of friendship, and antipathies simply results from a general response tendency in nominating others (reflected both in like and dislike choices). In other words, having more or fewer friends neither increased or decreased the likelihood of forming antipathies, and vice-versa.

While visibility and status theories assume that children need to have a general knowledge about which peers are more salient in their classroom (taking into account both friendship and antipathy ties) and act accordingly, balance implies higher cognitive requirements. Balance theory (Heider, 1946; see also Cartwright and Harary, 1956; Newcomb, 1961) assumes that actors are knowledgeable of each other's ties (positive and negative) and act accordingly by creating or breaking ties in or order to achieve balance (make the positive tie between two actors consistent with their negative ties with the third member of the triad). One could argue that the transitory nature of friendship and (specially) antipathy ties in preschool impairs this knowledge and prevent children to create (or maintain) balanced triads.

Children in general, and young children in particular, are very inaccurate when identifying the peers that dislike them (Bellmore and Cillessen, 2003; Neal et al., 2016). Agonistic exchanges between preschool children are much less frequent than exchanges of affiliative behaviors (Vaughn and Santos, 2009). Less exposure to this category of social interactions (Strayer et al., 1978) and the fact that children may withheld their negative views of one another (Bellmore and Cillessen, 2003), combined with the transitory nature of antipathies in preschoolers could explain why children have difficulties in identifying the peers that dislike them, or that dislike one another, and reacting to this knowledge. This may even explain the existence of few reciprocal antipathetic relationships (more common in older samples). An inaccurate picture of antipathetic relationships might also be a consequence of how social networks are encoded in memory. Networks are likely encoded as sets of triads (Brashears and Quintane, 2015); triadic closure being absent in antipathy networks might be an additional obstacle for preschool children social knowledge of dislike relationships.

Balance, status, and visibility theories are agency-based accounts of network structure where actors are expected to make choices influenced by their understanding of the network. Our results suggest that contrary to older children, preschoolers might not possess the cognitive capacity to encode their peer social network (i.e., who is liked or disliked by whom) and react to it, despite having highly structured relationships (i.e., organized relationships within functionally distinct subgroups; Daniel et al., 2015; Santos et al., 2015). Likely, the developmental dynamics of preschool children social networks involve minimal cognitive requirements. As detailed in Schaefer et al. (2010), to develop reciprocal and closed triadic friendships, and unequally allocate their preferences among existing peers, preschool children need: (a) only to be aware of their peers' behavior toward them and respond in kind (reciprocity), (b) use the increased propinquity of individuals who share mutual friends to interact with new peers (triadic closure), and (c) seek others based upon individual characteristics which are themselves unevenly distributed (in-degree popularity). Similarly, unequally distribution of antipathies (concentrated in few children) can simply result from the identification of undesirable individual characteristics that become more salient to the group as time goes by van den Oord et al. (2000).

Another possibility to explain the lack of dependence of friendship and antipathy network dynamics, that does not imply inaccurate perceptions of peers' relationships, would be to consider that preschool children have not yet developed shared norms to deal with antipathetic relationships (see Rakoczy and Schmidt, 2012 for a review on the ontogeny of social norms). For example, norms that would prevent children to befriend highly disliked peers, or befriend those disliked by their friends. Previous findings show that shared norms become more relevant as children grow older (Gifford-Smith and Brownell, 2003), evolving possibly as a way to stabilize group coordination and cooperation (Rakoczy and Schmidt, 2012). These social norms create more complex group dynamics where the co-development of friendship and antipathies can be expected.

### Limitations of the study

One should bear in mind that using different criteria to identify network ties (either from observational or sociometric data) creates networks with different densities. For example, our study reports a mean number of friends (degree) of ~4, Berger and Dijkstra (2013), of ~2.5, Rambaran et al. (2015) of ~8, and Schaefer et al. (2010) of ~6. There are some evidence that network size and density have bounding effects on network features (Faust, 2010); whether or not different network sizes and densities produce different model estimates, that could lead to different interpretations about the social processes behind tie formation, is still unknown. In spite of such circumstances, it does not seem to be the case for friendship networks where different studies have consistently found similar effects. Snijders and Baerveldt (2003) described a meta-analytical procedure that could be used to combine estimates from different samples (albeit, all models have to use the same effects) and test whether individual parameter estimates differ across networks. If differences occur, individual estimates could be further regressed on a set of variables (e.g., classroom size, density, sociometric data vs. observational data) to understand possible causes of variation (Lubbers, 2003).

One limitation of this study derives from the low stability found in peer relationships. Although low stability did not affect model convergence, stochastic actor-based models work best when stability is higher. Future studies should collect more data points within the same school-year to extend the findings presented here. With more data points it would be possible to observe more detailed developmental trajectories and to control possible stability related issues. Also, because higher developmental levels associate with more structured relationships (van den Oord et al., 2000; Daniel et al., 2015), future studies should also include specific indicators of development for each child and test how these indicators related with different network effects, particularly those dealing with the association of friendship and antipathy ties.

Although there is some discussion as to whether sociometric choices reflect true relationships (Strayer, 1980), given we were able to replicate previous findings dealing with network dynamics of friendship relations inferred from observational data (Schaefer et al., 2010), at least for friendships we are reasonably confident that our friendship networks do represent true relationships (see also Snyder et al., 1996, van den Oord et al., 2000 and Vaughn et al., 2000 for evidence supporting this claim). As to the dynamics of antipathy networks, there is no similar study using observational data to compare with. It would be extremely valuable to replicate our study using friendship and antipathy data both inferred from observational data.

Despite these limitations, this is the first study to address the co-evolution of friendship and antipathy networks in preschool children and in our view makes an important contribution to the literature. Our results show that different processes influenced the dynamics of ties in both networks and suggest that multivariate dependencies only play a more significant role in the peer group context in older children. We hope we are able to show that a combination of different sociometric tasks and the use of stochastic modeling approach can provide new nuances to the study of preschool children peer groups. Future studies should harness the potential of these models and sociometric data of preschool children to study the impact of social selection and social influence mechanisms on developmental outcomes of preschool children.

## Author contributions

JD, AS, BV: conception of the work. JD, MA, MF: acquisition of data. JD: data analysis. JD, AS, MA, MF: interpretation of data; drafting the manuscript; final approval of the version to be published; agreement to be accountable for all aspects of the work.

## Funding

This study was funded by FCT and NSF grants: PTDC/PSI/66172/2006, SFRH/BD/69863/2010, SFRH/BPD/82522/2011, SFRH/BD/80977/2011, UID/PSI/04810/2013, BCS 12-51322.

### Conflict of interest statement

The authors declare that the research was conducted in the absence of any commercial or financial relationships that could be construed as a potential conflict of interest.
