# Supplementary material for: Co-evolution of Friendships and Antipathies: A Longitudinal Study of Preschool Peer Groups
Source: Front Psychol. 2016 Oct 6;7:1509. doi: 10.3389/fpsyg.2016.01509 (PMC5052272; doi:10.3389/fpsyg.2016.01509)
Supplement: Supplementary file 1 [file DataSheet1.pdf]

Supplementary Material

Co-evolution of positive and negative networks: A longitudinal study of preschool peer groups

João R. Daniel[*], António J. Santos, Marta Antunes, Marília Fernandes, Brian E. Vaughn

[*] Correspondence: jdaniel@ispa.pt

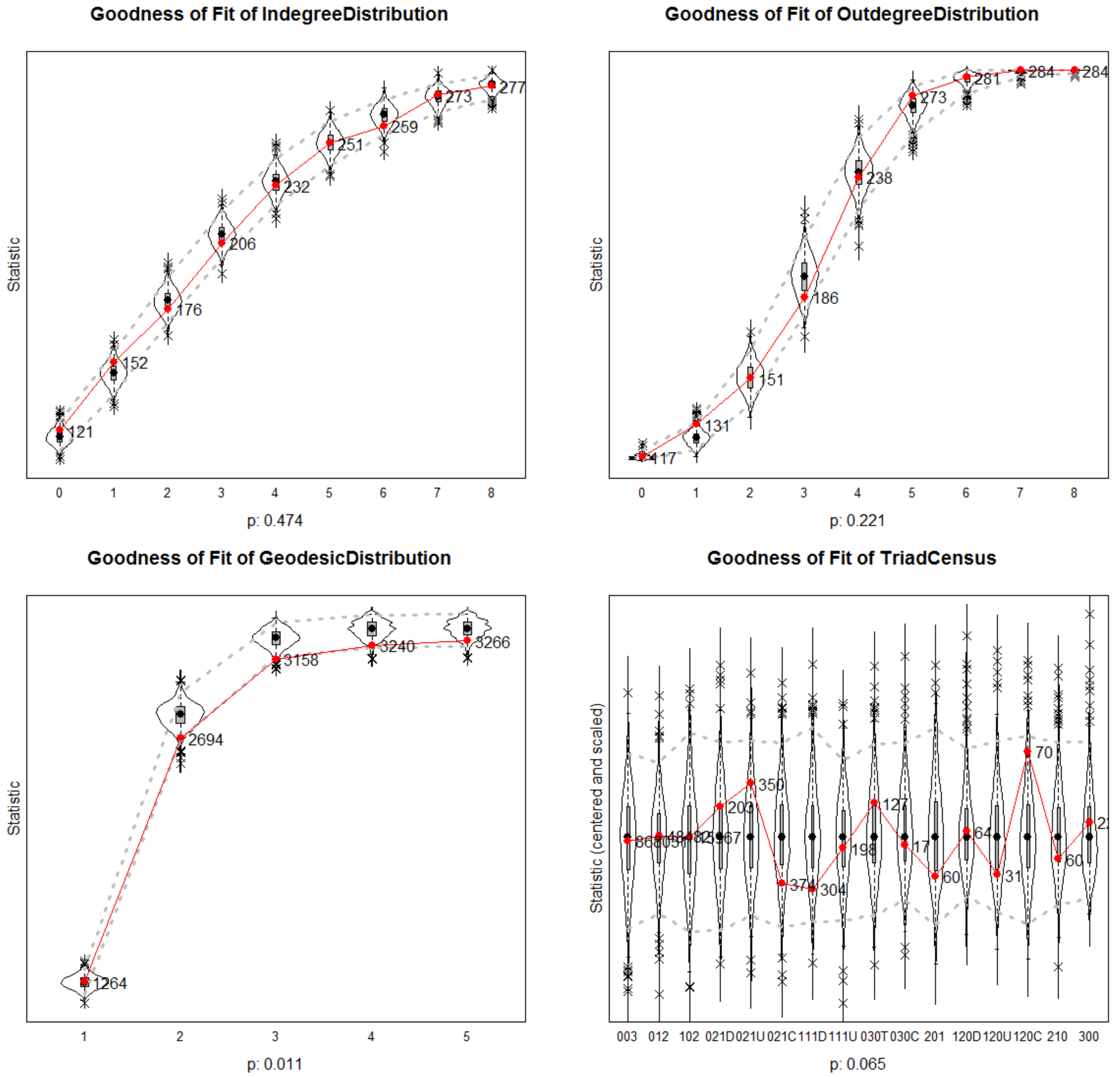

Supplementary Figure 1. Goodness of fit statistics for the univariate friendship network model

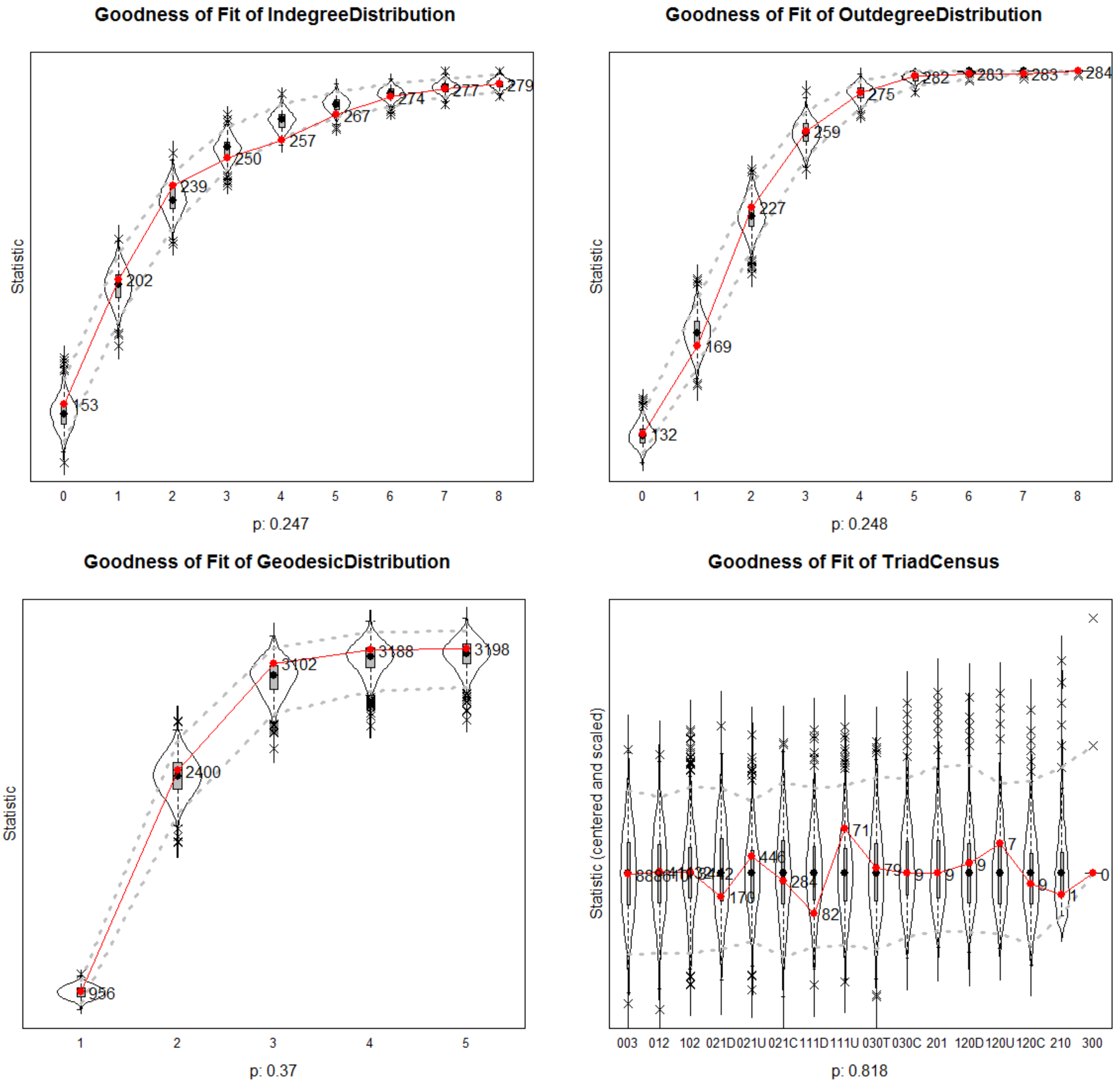

Supplementary Figure 2. Goodness of fit statistics for the univariate antipathy network model

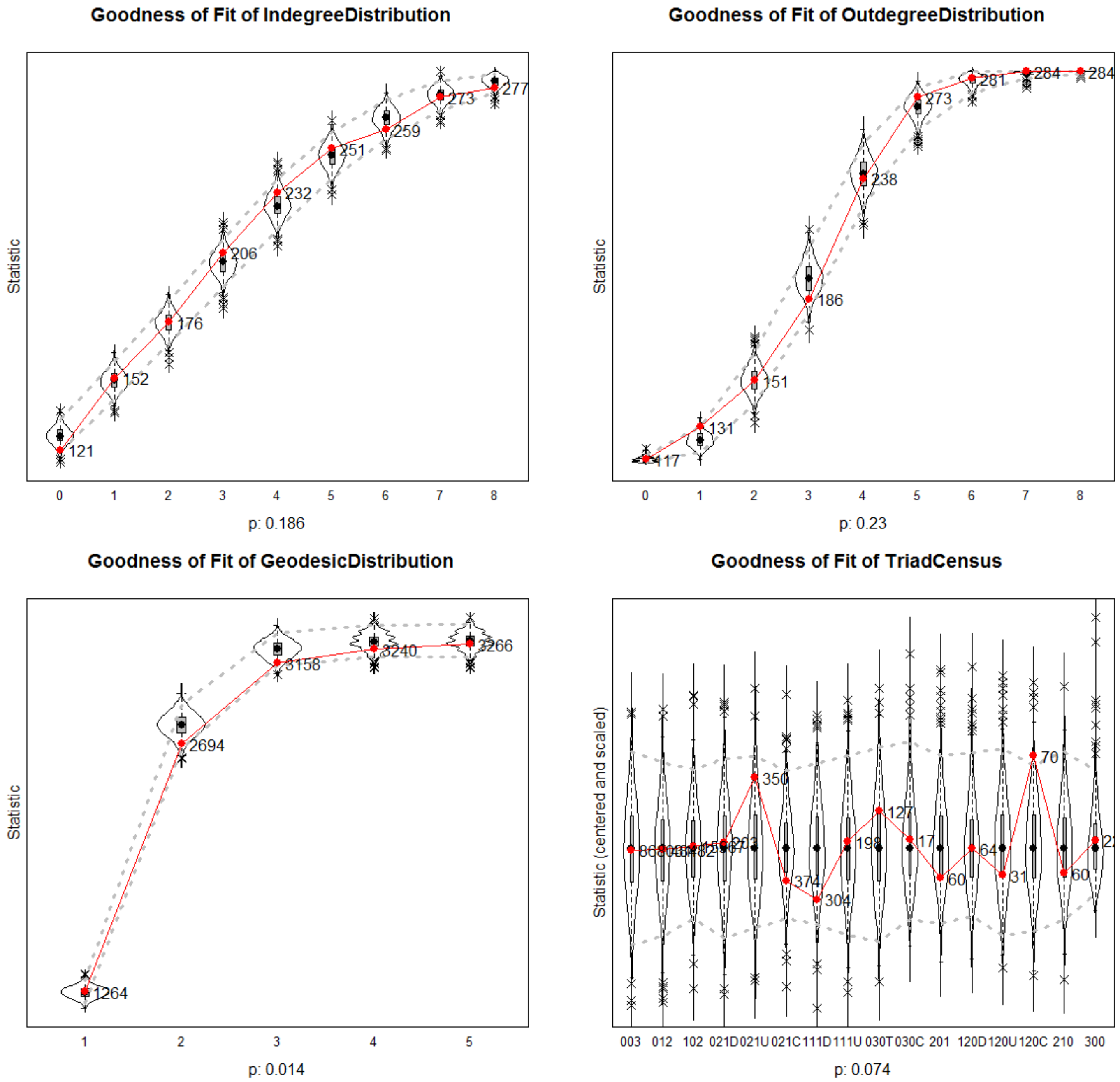

Supplementary Figure 3. Goodness of fit statistics for the multivariate model: Friendship network

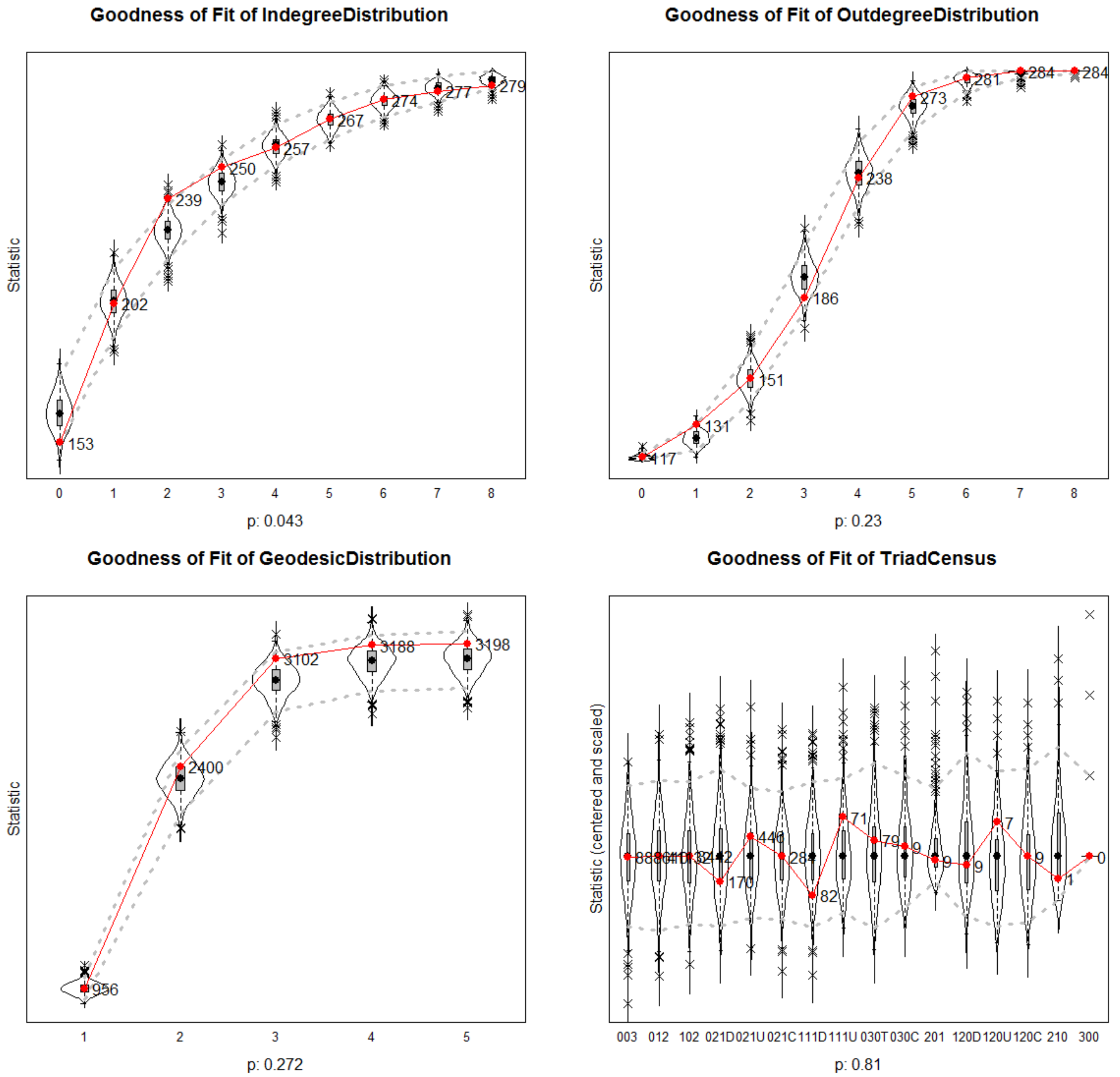

Supplementary Figure 4. Goodness of fit statistics for the multivariate model: Antipathy network

Supplementary Table 1. Multivariate triadic estimates (*β*) and standard Errors (SE) in simplified multivariate models

**Parameter**	***β***	***SE***
Degree-related network effects		
17. Friendship popularity on antipathy popularity (-)	-.12	.07
18. Antipathy popularity on friendship popularity (+)	-.13	.10
19. Friendship activity on antipathy activity (-)	-.05	.17
20. Antipathy activity on friendship activity (+)	-.01	.08
Triadic network effects		
21. Friends agreement (-) .	.31	.28
22. Reinforced animosity (-)	.13	.12
23. Shared enemy (+)	.23	.17
24. Enemy of my enemy #1 (+)	-.08	.30
25. Enemy of my enemy #2 (+)	.37	.38
26. Forced friends (+)	-.09	.20

Note. For brevity only multivariate effects' estimates are presented; estimates came from 10 different models (only one multivariate triadic effect per model); + and – signs following multivariate effects' names indicate whether friendship (+) or antipathy (-) ties were considered the dependent tie;; overall maximum convergence ratios < .25 and *t* statistics for deviations from targets < .10 in absolute value for all models; all *p* values of the joint significance test of time heterogeneity were *ns*.
